# Strengthening resources through identity-reframing interventions: empowerment for students with low socioeconomic status and with ADHD symptoms

**DOI:** 10.3389/fpsyg.2025.1525850

**Published:** 2025-06-25

**Authors:** Julius Möller, Stefan Reiss, Eva Jonas

**Affiliations:** Department of Psychology, University of Salzburg, Salzburg, Austria

**Keywords:** resources, social cure, social belonging, socioeconomic status, ADHD

## Abstract

**Introduction:**

Students draw on multiple resources to pursue a higher education degree. Vulnerable student groups, such as those from low socioeconomic status (SES) or with symptoms of Attention Deficit Hyperactivity Disorder (ADHD), may have fewer resources at their disposal. An essential resource in higher education settings is social belonging among their peers within the academic environment. Students from low SES or with ADHD symptoms might lack this sense of belonging due to their different background or their additional needs.

**Methods:**

Based on the social cure approach, we investigate two identity-reframing interventions (*N* = 392) that foster social belonging (Study 1) and relatedness need satisfaction (Study 2) and support academic engagement through reframing deficit narratives.

**Results:**

In Study 1, we found that the identity-reframing intervention successfully buffers the negative effect of low SES on social belonging, which in turn is linked to a higher academic self-concept and reduced fear of errors. In Study 2, the identity-reframing intervention mitigated the detrimental effect of ADHD symptoms on relatedness satisfaction. Further, we found that the intervention reduces the effect of ADHD symptoms on intrinsic motivation via academic identity.

**Discussion:**

We discuss how identity-reframing interventions foster social belonging and, in the context of social cure, positively influence academic engagement. The findings suggest that support programs for vulnerable student groups could be more effective if they emphasize the strengths gained from contending with past challenges, transforming these into valuable resources in higher education.

## 1 Introduction

Pursuing a university degree demands significant time and effort. Students must show considerable resilience and perseverance to complete their studies. They face multiple academic challenges on the way to attaining a tertiary education degree, from enrolling at a university and trying to understand how a university operates, to passing complex tests and assignments, to completing their final thesis. Not only does navigating academic obstacles take effort and time, but these academic challenges are accompanied by changes to students’ social lives. When starting university, students often relocate away from their familial home for the first time, putting themselves in a new environment on and around campus. Students now must organize their lives independently and cannot rely on their parents’ immediate support. To overcome these diverse challenges and complete their studies, they must access and effectively utilize various resources (Stelnicki et al., 2015).

Challenges are manifold and come from all kinds of individual and structural factors. In the groups we have specifically focused on, one common denominator is the challenge of feeling a sense of belonging to the academic world and their peers. Thus, we assume that students who feel they have more in common with the “prototypical “student experience more sense of belonging to the academic environment. Notably, students from vulnerable groups, such as those with low socioeconomic status (SES) or with learning disabilities, may face greater challenges due to their potentially limited preexisting resources (Jury et al., 2017; Sedgwick, 2018). Students with low SES are often portrayed from a deficit perspective. The narrative may include aspects such as a perceived lack of skills required for a higher education degree and insufficient financial support that makes them feel out of place at university, ultimately painting a picture of a struggling student. Previous meta-analyses by Liu et al. (2020) and Sirin (2005) demonstrated that low socioeconomic status is associated with low academic achievement. Consequently, low SES students are more likely to drop out of university than their high SES peers (Vignoles and Powdthavee, 2009). Low SES students face more psychological barriers than their high SES colleagues: They report more emotional distress and see themselves as less competent (Jury et al., 2017). Furthermore, students with low SES might lack the financial resources to participate in the same activities as their peers or have up-to-date technical equipment, thus being forced to work off campus (Devlin and McKay, 2018; Jury et al., 2019). These experiences might evoke a feeling of not belonging to their peers (Jetten et al., 2008).

Similarly, students whose learning is impacted by conditions like ADHD (attention deficit hyperactivity disorder) represent a group that faces challenges in higher education. Students with ADHD symptoms often underachieve academically compared to their peers (Henning et al., 2022), attaining a lower GPA and demonstrating slower progress toward graduation (DuPaul et al., 2021), which may lead to not finishing on time (Pope, 2010). These challenges could stem from lower organizational skills (Weyandt et al., 2013) or difficulties in social interaction with peers and university staff (Godfrey-Harris and Shaw, 2023). As a result, they may encounter more obstacles in gathering all the necessary information to navigate academic life successfully compared to their neurotypical peers. Consequently, they report lower self-esteem (Shaw-Zirt et al., 2005), feelings of being different, and experiencing a lack of social belonging, questioning their place within the student community (Godfrey-Harris and Shaw, 2023; Oram et al., 2023).

We acknowledge the vastly different challenges that students in both groups might face. A common experience for both groups, however, seems to be a feeling of not belonging to the student community. The feeling of not belonging to a relevant group has detrimental consequences not only for well-being but also for academic outcomes (O’Keeffe, 2013). We assume that fostering students’ awareness about beneficial, existing resources may lead to increased social belonging. This might be especially powerful for vulnerable student groups, like students from low-SES backgrounds or with ADHD symptoms.

In the present research, we investigate the impact of resource-strengthening, identity-reframing interventions on two vulnerable student groups: low-SES students and students with ADHD symptoms. We examine how these interventions affect students’ social belonging and influence motivation, academic self-concept, and fear of errors at university.

### 1.1 Studying as a resource-oriented process

A valuable framework for assessing students’ resources is the Conservation of Resources Theory (COR; Hobfoll, 1989). COR postulates that “individuals (and groups) strive to obtain, retain, foster, and protect those things they centrally value” (Hobfoll et al., 2018, p. 307). Four types of resources are distinguished: (1) Objects that might be resourceful due to their physical attributes or rarity, such as appropriate housing; (2) Conditions that include social support and a stable environment; (3) personal characteristics that help deal with stress, including certain personality traits. (4) Energies such as money, time, or knowledge, that are critical for gaining other resources, objects, or conditions.

The theory is grounded in four main principles: (1) Individuals are more sensitive to resource losses than gains. (2) Individuals must invest in their resources to be protected against resource loss. (3) The acquisition of resources becomes more prominent during periods of resource depletion. (4) When individuals’ resources are strained, they become defensive, protecting the self in potentially irrational ways.

How does the Conservation of Resources Theory (COR) apply within the context of higher education and the challenges that students encounter throughout their academic pursuits? COR tries to explain individuals’ ways of coping with stress, a prevalent experience for students on their path to their academic degrees. According to COR, stress arises when resources are threatened, lost, or when significant effort fails to acquire new resources (Alarcon et al., 2011). To understand that mechanism, it is vital to clarify how the four types of postulated resources can be applied in higher education. At university, object resources encompass, for example, appropriate housing as a vital resilience resource. It provides students with a room for their studying and necessary regeneration (Card and Thomas, 2018). Condition resources include social support and the feeling of belonging to a group. Both social support and a sense of belonging are significant predictors not only of academic achievements but also of well-being during their studies (O’Keeffe, 2013). Personal resources for higher education can include traits such as conscientiousness and persistence, enabling students to navigate academic challenges (Trapmann et al., 2007). Lastly, energy resources, such as time and financial capital, play a crucial role. For most students, pursuing a university degree is a substantial investment of time and effort but, foremost, money. Therefore, being able to afford a college education is a non-negotiable prerequisite for pursuing a tertiary degree. However, what happens when students’ resources are threatened or lost?

#### 1.1.1 Resources for students with low socioeconomic status

The academic journey can become exceedingly demanding when individuals have limited access to essential resources, such as those belonging to disadvantaged groups within higher education. A notable group of students who might perceive tertiary education as more challenging or threatening are students with low socioeconomic status (Devlin, 2013; Jury et al., 2017).

Low-SES students may differ significantly in object resources; for instance, they might still live at their parents’ home, maybe even sharing a room due to financial constraints that prevent their families from affording larger accommodations. Moving out might not be an option due to the financial pressure. Other object resources, such as laptops or means of transportation necessary for commuting to university, possibly cannot be accessed (Reisdorf et al., 2020). This might further differentiate their experience from their peers. Moreover, low-SES students often experience deficiencies in condition resources. Due to their contrasting backgrounds, they feel out of place at university and struggle to identify with their peers, leading to fewer personal relationships and, consequently, a diminished sense of belonging (Jetten et al., 2008). Research indicates that these are significant predictors of academic success (Walton and Cohen, 2011). Finally, low-SES students often face fewer energy resources, mainly regarding financial capital. Low-SES students, by name, have fewer financial resources than their peers, which might inhibit their ability to access necessary object resources and feel a sense of belonging to the university.

In sum, low-SES students seem to have more threatened resources than their peers. Thus, according to COR, they feel more stressed, resulting in a defensive and self-protective state. This might further hamper their sense of belonging at university.

#### 1.1.2 Students with ADHD symptoms

Another group that encounters difficulties in higher education comprises students with learning disabilities, with attention deficit hyperactivity disorder (ADHD) being one of the most prevalent. Students diagnosed with ADHD often struggle to concentrate on a single task at a time and experience chronic inner restlessness, resulting in significant difficulty when faced with situations that require them to sit quietly or wait for their turn (Sedgwick, 2018). These symptoms contribute to a more complex university experience for ADHD students compared to their peers (Meaux et al., 2009).

From the perspective of COR, students with ADHD may find themselves with more threatened resources compared to their neurotypical peers. Unlike students from low-SES backgrounds, whose resource limitations may stem from financial constraints (energy resources), the challenges faced by students with ADHD may be primarily related to conditions and personal resources. ADHD students face unique challenges at university, such as requiring additional time to complete exams or difficulties maintaining attention during lectures (Advokat et al., 2011). These additional needs may result in them feeling different from their peers, thus hampering their sense of belonging within the student community (Godfrey-Harris and Shaw, 2023). To successfully pursue a tertiary degree, however, they might have to address these needs and ask university staff for additional time or other forms of support (Miller et al., 2015). This may result in a dilemma as openly acknowledging these needs may further aggravate the feeling of not belonging to their peers. In sum, for students with ADHD symptoms, their resources of belonging to their peers might be especially threatened, leading them to feel isolated and maybe even self-protective (Jong et al., 2024). This isolation might further hinder their access to other resources and impede their success in the university environment.

Students from disadvantaged groups like low-SES students or students diagnosed with ADHD might have their resources threatened, obstructing them from coping with academic challenges and even evoking stress. Notably, the resources of belonging to a group seem to be particularly compromised for these vulnerable student groups. But why is it beneficial to feel part of a group to cope with threats and challenges effectively?

### 1.2 Social cure

A shared group membership has various positive implications for individuals (Jetten et al., 2017). The sense of belonging fulfills basic psychological needs and thus serves as a psychological resource (Baumeister and Leary, 1995). Moreover, group membership leads to individuals feeling a sense of belonging, meaning, and control in life. Taking together, being part of a group makes people feel healthier and enhances their well-being. This beneficial effect of social identification and group membership is called social cure (Jetten et al., 2012). Fostering students’ sense of belonging, despite additional challenges or negative narratives, is essential. By establishing a sense that everyone—regardless of their difficulties and weaknesses—is part of the student community and can contribute their unique strengths, the social cure effect can be activated. One might assume that this effect only applies to identification with groups that are positively associated, presumably those with high control, power, or simply being in the majority. This reasoning seems valid, as we know that group membership does not only have positive consequences. If a group such as refugees, members of the LGBTQ community, or religious minorities is stigmatized, belonging to these groups can also bring harm. These individuals face prejudice, racism, and discrimination (Esses, 2021; Herek and McLemore, 2013; Rehman and Hanley, 2023). This leads to lower psychological well-being and health and may even prompt members to distance themselves from their groups (Schmitt et al., 2014; Schmitt and Branscombe, 2002).

#### 1.2.1 Rejection identification model

However, a growing body of research challenges the assumption that identification with disadvantaged groups leads to reduced well-being and distancing. Numerous studies within the context of social cure indicate that social identification can be beneficial for people who belong to stigmatized or marginalized groups, such as refugees, immigrants, or minorities (Aybar Camposano et al., 2024; Cruwys et al., 2014; Muldoon et al., 2017; Skrodzka et al., 2024). The Rejection Identification Model (RIM; Branscombe et al., 1999) provides a framework for understanding the psychological benefits of identifying even with a disadvantaged group. According to the RIM, being a member of a disadvantaged group often results in exposure to prejudice, which understandably diminishes individual well-being. However, RIM posits that being part of a stigmatized group also reinforces identification with that group. This may be due to the need to belong, which might only be possible with the disadvantaged ingroup in the face of prejudice and discrimination (Branscombe et al., 1999). This heightened ingroup identification, even with a disadvantaged ingroup, can act as a protective factor, positively influencing well-being, or in other words, providing the social cure effect. In line with the social cure approach and aligned with the Rejection Identification Model, we propose that ingroup identification acts as a resource for stigmatized groups in higher education, such as students from low SES backgrounds and those exhibiting ADHD symptoms.

#### 1.2.2 Social cure for students with low socioeconomic status

The social cure approach can also be applied to the context of higher education. Research indicates that, for example, social identification buffers feelings of loneliness at university (McIntyre et al., 2018). In higher education, a sense of belonging to a group fosters psychological well-being and, importantly, academic motivation and a reduced intention to drop out (Gillen-O’Neel, 2019). For instance, Freeman et al. (2007) demonstrated that students’ sense of belonging was associated with greater self-efficacy and intrinsic motivation. Similarly, Pedler et al. (2022) found positive relationships between belonging, motivation, and enjoyment in the academic setting. Interestingly, they found a lower sense of belonging in first-generation students – those whose parents do not hold a tertiary degree – than students from academic households (i.e., continuous-generation students). First-generation students are often depicted as a disadvantaged group in higher education due to their lack of social capital, such as knowledge and information acquired through social interaction, compared to their peers (Robison et al., 2002; Soria and Stebleton, 2012). This effect can also be found in other disadvantaged groups in the university environment. For example, first-year students from ethnic minorities report a lower sense of belonging than their white peers (Johnson et al., 2007). Likewise, Trawalter et al. (2021) show that students from low socioeconomic backgrounds experience lower social belonging at university.

#### 1.2.3 Social cure for students with ADHD symptoms

Attention deficit hyperactivity disorder and low-SES students may face different challenges in their academic journey that ultimately result in similar threats to their social belonging at university. Whereas low-SES students struggle more with acknowledging that they stand under significantly more financial pressure than their peers, students with ADHD may feel out due to a different reason. Students with ADHD may experience more challenges than their neurotypical peers, leading them to question whether they can meet the demands of higher education (Oram et al., 2023). This could weaken their sense of belonging, which in turn makes them feel out of place. In contrast to low-SES students, this might not be the case because of their social background but because of their perceived academic inadequacy.

When students with ADHD symptoms compare themselves to their neurotypical peers, they may feel disadvantaged in terms of academic performance, organization skills, and the ability to adhere to institutional norms in academia (Sedgwick, 2018). These unfavorable comparisons can undermine their academic self-concept, resulting in lower self-esteem and reduced academic achievement (Bodalski et al., 2023; Scholtens et al., 2013; Shaikh, 2018). This sense of disadvantage may cause them to internalize the belief that they are not “good students.” Thus, their motivation and commitment to their studies fade, further threatening their sense of belonging and reducing their likelihood of success.

Research supports this link between ADHD symptoms and academic challenges. Khalis et al. (2018) found that ADHD symptoms in students were positively related to internalizing difficulties, meaning they felt more anxious the more symptoms they reported. Additionally, while ADHD symptoms positively predicted their belonging to the university, they negatively affected students’ academic performance. Notably, there was a significant interaction between ADHD and reciprocated friendships for anxiety and belonging to the university. Students who experienced such friendships experienced lower anxiety and a higher attachment to university. Further, during the COVID-19 pandemic, students with ADHD experienced more loneliness than their neurotypical peers. Laslo-Roth et al. (2022) found that the relationship between ADHD symptoms and loneliness was mediated by learning difficulties and, importantly, peer and faculty support. In summary, social belonging is vital in shaping the higher education experience for students with ADHD symptoms. Feeling as part of a group or receiving social support seems to buffer the effect of ADHD symptoms on academic engagement.

### 1.3 Identity-reframing interventions

Both low-SES and ADHD students appear to have distinct yet comparable narratives in higher education. Both groups share that they tend to underachieve academically (Henning et al., 2022; Jury et al., 2017). Importantly, through the lens of social cure, both groups face the struggle of feeling excluded from the student community, leading them to view themselves as different from their peers (Godfrey-Harris and Shaw, 2023; Jetten et al., 2008). While these aspects seem to unite their experiences, the narratives explaining why this occurs differ significantly. Whereas low SES students are more likely to face social identity threats stemming from their socioeconomic backgrounds (Bauer et al., 2025), the narrative for ADHD students often revolves around their academic performance, which can lead to feelings of inadequacy regarding the abilities required to succeed in an academic environment (Stevens et al., 2022). Although the roots of a lack of belonging in higher education differ between low SES students and those exhibiting ADHD symptoms, they share a common narrative of being perceived as the odd one out and not fitting into university.

According to COR, belonging to a group serves as a crucial psychological resource (Hobfoll, 1989). Low-SES students may lack object resources or financial resources they can rely on; thus, feeling like they are part of the student community seems to be an ever-so-important psychological resource for them to succeed (Bauer et al., 2025). For students with ADHD symptoms, this situation may be comparable. These students may question their academic competencies and thus doubt their status as qualified students, making them feel out of place within the student community (Stevens et al., 2022). Therefore, for students with ADHD symptoms, a strong sense of belonging to this community may help them buffer the negative effects of ADHD on their academic experiences.

To enhance the sense of belonging to the student community, altering the deficit narratives surrounding these groups can be promising. Negative narratives can become self-fulfilling (Bauer et al., 2025), indicating that established narratives lead students with low SES and ADHD to act in accordance with these assumptions, resulting in an even greater deficit in social belonging. Even more concerning is the possibility that these narratives obscure the strengths arising from their individual backgrounds. Evidence indicates that low SES students demonstrate great resilience or cooperation that might be beneficial in higher education (see Hernandez et al., 2021). Students with ADHD may excel in tasks that require creativity or swift cognitive shifts. However, the deficit narrative often overshadows these strengths, meaning they are not available for the students. A common social psychological intervention to counteract this phenomenon for disadvantaged groups associated with deficit-based narratives involves emphasizing strengths specific to their backgrounds (Bauer et al., 2021, 2023; Hernandez et al., 2021). Hernandez et al. (2021) showed that by activating strengths that low SES students gain through their lived experience, they report increased academic persistence, self-esteem, and academic success. A more nuanced way to change deficit narratives about group members is through an explicit identity-reframing approach (Bauer and Walton, 2024). This identity-focused approach aims to shift the narrative from viewing oneself as a member of a disadvantaged group to recognizing oneself as a resourceful individual who has developed unique strengths by facing and overcoming challenges associated with their disadvantaged background. These strengths can empower individuals to tackle future challenges, even in different contexts. Bauer et al. (2021) found that reframing challenges as sources of strength led to increased academic engagement among refugees. Furthermore, this approach can also be applied to low SES students, where Bauer et al. (2025) demonstrated that this identity- based narrative- changing approach boosted low SES students’ performance on an academic task and showed longitudinal effects by raising their course grades.

However, there is no evidence that identity-reframing interventions increase social belonging among disadvantaged groups in higher education. As a sense of belonging is a crucial resource for students and a unifying element in the deficit narratives of low SES students and those with ADHD symptoms, we believe it is essential to advance research on identity-reframing interventions in this context.

### 1.4 The present research

In this study, we try to foster academic engagement of members of disadvantaged groups by using brief identity-reframing interventions focusing on increasing social belonging. In the first study, we will implement an identity-focused, background-specific strength intervention emphasizing how mastering unique challenges due to their socioeconomic background helps them navigate the academic demand at university. According to the social cure approach, this intervention should foster well-being by reframing negative attributes attached to their ingroup and posing them even as possible resources. We expect this to increase ingroup identification, which is related to them feeling more in place in the academic context. Ultimately, this identification should lead to more academic engagement.

In the second study, we will address students with ADHD, a group often stigmatized in higher education settings. We will also employ an identity-reframing intervention to challenge the deficit narrative associated with ADHD and to strengthen their ingroup identification in the sense of a social cure. We hypothesize that this intervention will positively predict academic engagement in the form of intrinsic motivation.

With the current research, we aim to contribute theoretically by extending the literature on social cure and identity reframing. To our knowledge, no existing studies have combined the social cure and identity-reframing approaches. We seek to demonstrate that by altering deficit narratives about stigmatized groups and highlighting members’ strengths gained from overcoming unique challenges, ingroup identification can be enhanced. This would offer an innovative perspective on the identity-reframing literature. Regarding social cure, we aim to show that this increased identification directly relates to students’ academic well-being, including intrinsic motivation and engagement in higher education. Importantly, by focusing on students exhibiting symptoms of ADHD, we target a group that, to date, has not been well explored in the social cure and identity reframing literature. Furthermore, our research has practical implications as well. If our assumptions regarding the critical role of social belonging for these marginalized groups are accurate, it will encourage the design of support programs that specifically focus on fostering a sense of belonging among them. Moreover, if reframing the narrative proves to be impactful, it can prompt universities to reflect on and adapt their communication strategies with these vulnerable groups accordingly.

## 2 Study 1

### 2.1 Introduction

This study aims to mitigate the impact of being a low-SES student, an academically disadvantaged group, on academic engagement by emphasizing background-specific strengths. In this case, we will examine academic self-concept and fear of errors, both relevant predictors of academic success (De Castella et al., 2013; Wu et al., 2021). “Academic self-concept refers to individuals’ knowledge and perceptions about themselves in achievement situations” (Bong and Skaalvik, 2003, p. 6). Thus, the academic self-concept comprises all cognitive representations of one’s abilities in performance situations in the academic context. Research shows that the more students see themselves as capable, the better they perform in achievement situations (Marsh and Martin, 2011). Being a student with a low-SES background makes it more likely to have a lower academic self-concept, potentially leading to reduced academic engagement (Chevalère et al., 2023).

On the other hand, fear of errors involves the apprehension of making mistakes or failing in achievement setting and, if possible, avoiding these situations (Elliot and Sheldon, 1997). Importantly, students’ fear of errors or failure is linked with students’ motivation, self-esteem, behavior, and, subsequently, academic achievement (Choi, 2021). Evidence suggests a negative relationship between socioeconomic status and fear of errors, indicating that students with lower SES are likely to experience more fear of failure in academic settings (Sonnak and Towell, 2001).

In the present study, we assume that an identity-reframing, background-specific strength intervention can help improve student outcomes regarding academic self-concept and fear of errors, thus increasing their resources for academic success. In the sense of social cure, we expect that the intervention will lead to higher social belonging for low-SES students (H1.1), which in turn is associated with a higher academic self-concept (H1.2) and reduced fear of errors (H1.3). Exploratively, we will also investigate if the intervention fosters low-SES students’ academic identity, which is then related to a higher academic self-concept and diminished fear of errors.

### 2.2 Method

#### 2.2.1 Participants

We recruited 282 participants, with 11 participants failing the attention check; the final sample consisted of 270 participants. To participate in the study, students must be actively enrolled at university. On average, students were *M* = 23.51 (*SD* = 5.34) years old. Regarding gender, 212 (78.52%) identified as female, 57 (21.11%) as male, and one participant identified as diverse (0.37%). 137 (50.74%) participants stated their primary country of residence as Germany, 115 (42.59%) as Austria, and 18 (6.67%) participants reported other countries of residence. Only measures pertinent to the current research are reported.^1^

#### 2.2.2 Measures

##### 2.2.2.1 Social belonging

We measured social belonging with a five-item scale previously used by Möller et al. (2022). After analyzing the factorial structure, we excluded two items (for factor analysis, see Supplementary Table 1). Participants answered on a six-point Likert scale ranging from “do not agree at all” to “fully agree” (e.g., “I already have many good contacts with the other students at my department.”; *α* = 0.81).

##### 2.2.2.2 Academic identity

We assessed academic identity with a seven-item scale by Möller et al. (2022). However, after factor analysis, we excluded two items (for factor analysis, see Supplementary Table 1). Participants answered the statements on a six-point Likert scale ranging from “do not agree at all” to “fully agree” (e.g., “I can identify well with my studies.”; Cronbach’s alpha α = 0.78).

##### 2.2.2.3 Fear of errors

We measured students’ fear of errors in the university setting using an adapted scale from Spychiger et al. (2006). We adjusted the wording of the items to suit the university setting better as the original items are framed for the school context (e.g., “I’m often afraid of saying the wrong thing during my studies.”). Participants answered the statements on a six-point Likert scale ranging from “do not agree at all” to “fully agree” (α = 0.90).

##### 2.2.2.4 Academic self-concept

We assessed academic self-concept using a five-item scale (Dickhäuser et al., 2002). Participants answered the statements on a six-point Likert scale, with poles being adapted for each item (e.g., “In my studies, I can do…” [poles for this item: little – a lot]; α = 0.82).

##### 2.2.2.5 Subjective socioeconomic status

We measured the subjective socio-economic status of participants’ families using the Macarthur Ladder with ten rungs (Adler et al., 2000). Participants had to indicate on which rung they would place their family with the highest rung, which represented people who are highest up in society concerning money, education, and jobs. The lowest rung represented people with the least amount of money, the worst education, and no jobs or jobs with little prestige. The mean score was *M* = 6.21 (*SD* = 1.69).

#### 2.2.3 Procedure and design

Before taking part in the study, participants gave informed consent. After we assessed demographic information, including SES, participants were randomly assigned to either the experimental background-specific strength condition or the control condition. Students in the experimental condition had to write a short essay about the following questions. (1) “When you reflect on your childhood and youth, what specific challenges and difficulties did you face?” (2) “What positive resources, strengths, and skills can you draw from these personal challenges that will help you achieve your goals in your studies today?” With these prompts, we deviate from previous identity-reframing and background-specific strength interventions. Other studies offered a short introduction to the topic (e.g., first generation students) and presented stories of individuals who developed strengths from facing challenges unique to their group, before allowing participants to reflect on their own gained strengths (Bauer et al., 2025). We chose to skip the introduction and presentation of stories to make the intervention accessible to all students, regardless of their socio-economic background. We believe every student faces challenges in childhood and youth that can provide strengths in higher education. By skipping the focus on socio-economic status, we also aimed to avoid self-stereotyping for those from low SES backgrounds by not activating the deficit narrative. Moreover, this approach allows for quick adaptation to future contexts, as no prior interviews are necessary to write the success stories of other groups members. Participants in the control condition also had to answer part 1 but did not answer part 2, i.e., they also considered challenges and difficulties in childhood and youth. However, they did not reflect on how these experiences can be positive resources but on how much those still concern the participants today. Afterward, we measured academic identity, social belonging, academic self-concept, and fear of errors. Only variables pertinent to the manuscript are reported.

#### 2.2.4 Analyses

To assess whether the intervention indeed buffers the effect of SES on these resources, we will use a moderated mediation model. In this model, SES will function as the independent variable, with the resource, either academic identity or social belonging, as the mediator. The intervention will then moderate the path between SES and the resource. Academic self-concept and fear of errors are included as the dependent variables in the model (for full effects, see Supplementary Tables 2, 3, 6, 7).

### 2.3 Results

For an overview of means, standard deviations, and correlations of the variables, see Table 1.

**Table 1:** Correlation matrix of the variables in Study 1.

Variable	*M*	*SD*	1	2	3	4
1. SES	6.23	1.66				
2. Social Belonging	3.89	1.27	0.18** [0.06, 0.29]			
3. Academic Identity	4.67	0.88	0.03 [−0.09, 0.15]	0.18** [0.06, 0.29]		
4. Academic Self-Concept	4.21	0.76	0.03 [−0.09, 0.15]	0.23*** [0.11, 0.34]	0.37*** [0.26, 0.47]	
5. Fear of Errors	3.17	1.34	−0.06 [−0.17, 0.06]	−0.23*** [−0.34, −0.12]	−0.20** [−0.31, −0.08]	−0.39*** [−0.48, −0.28]

SES represents the subjective socioeconomic status. M and SD represent mean and standard deviation, respectively. Values in square brackets indicate the 95% confidence interval for each correlation. *p < 0.05, **p < 0.01, ***p < 0.001.

Correlations show that lower SES is associated with less social belonging at university. This can be evidence that being a member of a disadvantaged group in the academic setting leads to feeling out of place in higher education and consequently lacking social belonging as a psychological resource at university. Interestingly, SES and academic identity are not significantly correlated, indicating that being part of a disadvantaged group does not impact the way students see themselves as students.

#### 2.3.1 Manipulation check

We deviated from the identity-reframing interventions used by Bauer et al. (2025) by shortening the process. In the intervention condition, participants considered the challenges they confronted during childhood and adolescence, then identified strengths applicable to their studies derived from these experiences. In previous studies, students receiving a brief introduction on the topic, and then read stories in which they successfully developed strengths from experiences. Subsequently, they engaged in the same reflection task that we implemented. To evaluate whether shortening the intervention, and therefore avoiding stereotyping for low SES students, was effective, we coded the participants’ answers to the reflection task in both the intervention and control conditions. We classified the responses according to this framework (see Table 2): (1) strengths successfully derived from past challenges, (2) only challenges described without any specific strengths identified, (3) a mixture of strengths and weaknesses from previous challenges, (4) solely weaknesses reported, remaining with a deficit narrative, (5) responses that could not be categorized. A participant’s response in the intervention group that effectively derived strengths from previous challenges (1) reads as follows:

**Table 2:** Manipulation check of the intervention by qualitatively coding participants’ responses.

Category	Intervention condition	Control condition
1. Strengths successfully derived from past challenges	86.81%	7.14%
2. Only challenges described without any specific strengths identified	8.33%	35.71%
3. Mixture of strengths and weaknesses from previous challenges	3.47%	26.19%
4. Weaknesses reported, remaining with a deficit narrative	0.00%	24.60%
5. Responses that could not be categorized	1.39%	1.34%

*“Due to the sometimes financially difficult situation and my three siblings, I often had to take a back seat, share a lot, and my wishes could sometimes not be fulfilled or expressed. Unlike many of my classmates, I got less pocket money and did not have the latest fashion/clothes. […].*

*I am very grateful to my parents today, as I now realize what a challenge those times must have been. Gratitude also helps me in my studies as I do not take anything for granted. I get by on very little money, which is an advantage as a student. […].”*

An example of a participant’s response in the control condition that remained with the deficit narrative stemming from previous challenges (4) would be:

*“A major challenge was the financial insecurity that was a recurring theme, the lack of support from grandparents, and the constant confrontation with being a ‘working-class child’ with a migrant background. […].*

*“The financial worries, which have still not been resolved. For a long time, I doubted whether studying could make sense if you do not get any financial support from home.”*

#### 2.3.2 Social belonging as a resource

We used a moderated mediation model to test for the buffering effect of the identity-reframing intervention. In the first model, we used SES as the independent variable, academic self-concept as the dependent variable, and social belonging as the mediator. We found a significant path of SES on social belonging, *b* = 0.24, *SE* = 0.07, *p* = 0.001, 95% CI [0.10, 0.38], indicating that higher subjective socioeconomic status was associated with greater reported social belonging. There was no significant main effect of the intervention on social belonging, *b* = 0.00, *SE* = 0.15, *p* = 0.980, 95% CI [−0.30, 0.30].

**Figure 1:**
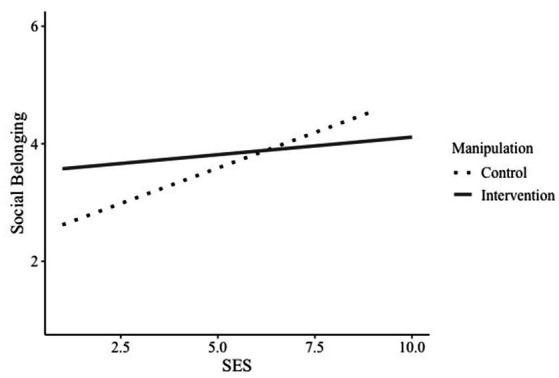
Interaction of manipulation and SES on social belonging. SES = Socioeconomic status.

We further found a non-significant trend for the interaction between SES and the intervention on social belonging, *b* = −0.18, *SE* = 0.09, *p* = 0.052, 95% CI [−0.36, 0.00] (H1.1). Conditional effects for the mediation showed that for the control group, there was a significant relationship between SES and social belonging, *b* = 0.24, *SE* = 0.07, *p* = 0.001, 95% CI [0.10, 0.28], such that students with higher subjective socioeconomic status reported more social belonging. However, for the intervention condition, there was no such significant path, *b* = 0.06, *SE* = 0.06, *p* = 0.321, 95% CI [−0.06, 0.18], suggesting that reflecting on background-specific strengths neutralized the influence of SES on social belonging (see Figure 1). When investigating the interaction at different levels of SES, results indicate that the effect of the intervention may be more beneficial for low SES students. It has to be noted that the Johnson-Neyman method revealed only a non-significant trend for those students (see Supplementary Tables 4, 5).

##### 2.3.2.1 Academic self-concept

There was no direct association between SES and academic self-concept, *b* = 0.00, *SE* = 0.03, *p* = 0.907, 95% CI [−0.06, 0.05]. However, social belonging was significantly associated with academic self-concept, *b* = 0.14, *SE* = 0.04, *p* = 0.001, 95% CI [0.07, 0.21], indicating that the more participants feel part of the student community, the more able they see themselves academically. As the interaction between SES and the intervention on social belonging showed a trend for significance, and social belonging was then positively related to academic self-concept, we tested for the indirect effect of the proposed moderated mediation model.

**Figure 2:**
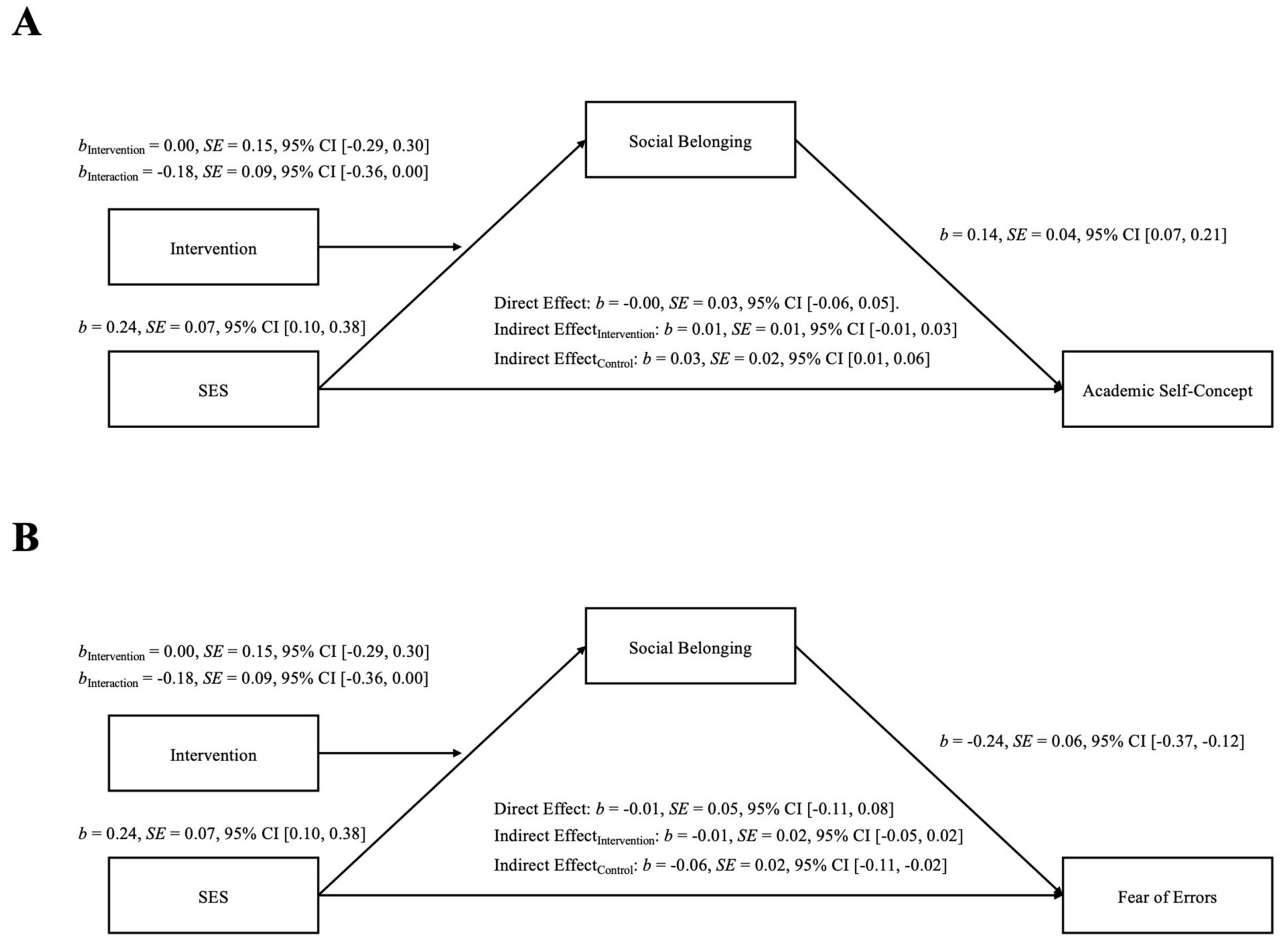
Moderated mediation models with social belonging as the mediator for **(A)** academic self-concept and **(B)** fear of errors. *B*-values indicate unstandardized regression coefficients. SES = Socioeconomic status.

For the control group, we found an indirect path of SES via social belonging on academic self-concept, *b* = 0.03, *SE* = 0.02, 95% CI [0.01, 0.06]. Importantly, this indirect path was absent for the intervention group, *b* = 0.01, *SE* = 0.01, 95% CI [−0.01, 0.03]. This shows that the identity-reframing intervention successfully altered the indirect effect of SES via social belonging on academic self-concept (H1.2). It has to be noted that the index of the moderated mediation was not significant, *b* = −0.02, *SE* = 0.02, 95% CI [−0.06, 0.00], see also Figure 2A.

##### 2.3.2.2 Fear of errors

We applied the same model for fear of errors as the outcome variable (see Figure 2B). Again, there was no significant direct association of SES on the outcome variable, *b* = −0.01, *SE* = 0.05, *p* = 0.795, 95% CI [−0.11, 0.08]. However, social belonging was negatively associated with fear of errors, *b* = −0.24, *SE* = 0.06, *p* = 0.001, 95% CI [−0.37, 0.12]. Thus, we again tested for a potential indirect effect of SES on fear of errors via social belonging. We found a significant indirect path via social belonging on fear of errors for the control group, *b* = −0.06, *SE* = 0.02, 95% CI [−0.11, −0.02], suggesting an indirect association of SES on fear of errors by social belonging. In other words, students with low-SES backgrounds report a higher fear of errors due to their lower sense of belonging. More importantly, in line with our assumptions, this indirect path did not appear for the intervention group, *b* = −0.01, *SE* = 0.02, 95% CI [−0.05, 0.02]. This indicates that an identity-reframing intervention buffers the negative association between SES and social belonging, which in turn is related to less fear of errors (H1.3). However, the index of the moderated mediation did not reach significance, *b* = 0.04, *SE* = 0.03, 95% CI [0.01, 0.11], see also Figure 2B. Again, due to the chosen model, we can only draw causal effects of the intervention on social belonging.

Overall, these findings suggest that by focusing on background-specific strengths and thereby reframing the deficit narrative of low SES students, these students are not dependent on social belonging to have a positive academic self-concept and feel secure when making errors. Instead, they now have the self as a psychological resource. Reflecting on their strengths makes them see their socioeconomic background not as a barrier to feeling integrated into the academic community.

#### 2.3.3 Academic identity as a resource

We tested the moderated mediation model for academic self-concept and fear of errors, this time using academic identity instead of social belonging as the mediator.

**Figure 3:**
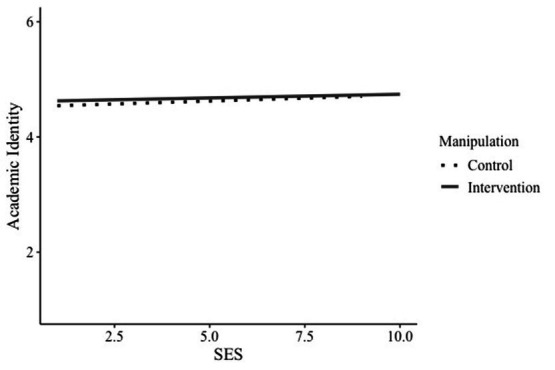
Interaction of manipulation and SES on academic identity. SES = Socioeconomic status.

Firstly, for the model predicting academic self-concept, we found no significant paths of SES, *b* = 0.02, *SE* = 0.05, *p* = 0.685, 95% CI [−0.08, 0.12], the manipulation, *b* = 0.04, *SE* = 0.11, *p* = 0.682, 95% CI [−0.17, 0.26], or their interaction academic identity on the mediator, *b* = −0.01, *SE* = 0.07, *p* = 0.908, 95% CI [−0.14, 0.12]. Thus, the identity-reframing intervention did not influence students’ academic identity (see Figure 3).

##### 2.3.3.1 Academic self-concept

**Figure 4:**
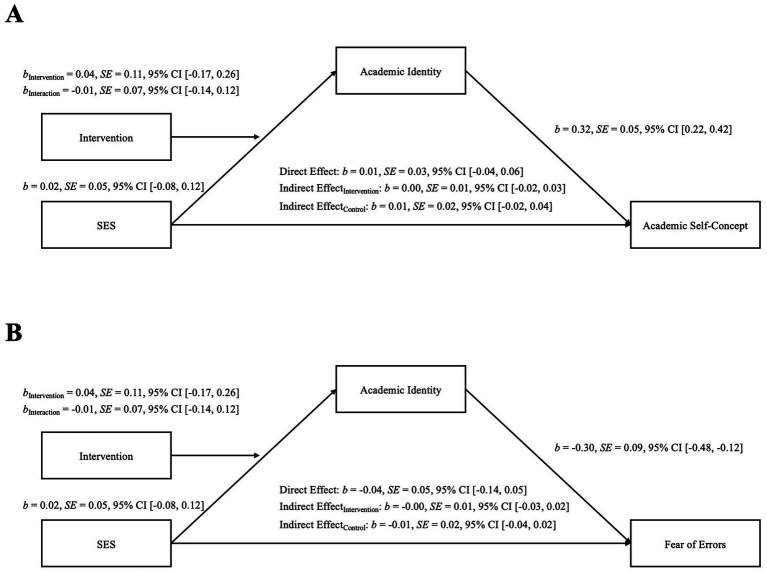
Moderated mediation models with academic identity as the mediator for **(A)** academic self-concept and **(B)** fear of errors. *B*-values indicate unstandardized regression coefficients. SES = Socioeconomic status.

We found no significant direct association between SES and academic self-concept, *b* = 0.01, *SE* = 0.03, *p* = 0.692, 95% CI [−0.04, 0.06]. However, academic identity was associated with academic self-concept, indicating that the more academic identity students reported, the more capable they perceived themselves in the academic environment, *b* = 0.32, *SE* = 0.05, *p* < 0.001, 95% CI [0.22, 0.42]. Thus, we tested for indirect paths of SES on academic self-concept via academic identity. For neither the control condition, *b* = 0.01, *SE* = 0.02, 95% CI [−0.02, 0.04], nor the intervention condition, *b* = 0.00, *SE* = 0.01, 95% CI [−0.02, 0.03], a significant indirect path of SES via academic identity on academic self-concept could be found. These findings suggest that the intervention did not significantly influence academic identity or, consequently, academic self-concept, see also Figure 4A.

##### 2.3.3.2 Fear of errors

There was no direct association of SES on fear of errors, *b* = −0.04, *SE* = 0.05, *p* = 0.399, 95% CI [−0.14, 0.05]. However, we found a significant path of academic identity on fear of errors, *b* = −0.30, *SE* = 0.09, *p* = 0.001, 95% CI [−0.48, −0.12], indicating that the more participants perceive themselves as students, the less fear of errors at university they report. When testing for indirect associations from SES on fear of errors via academic identity, neither the indirect path for the control group, *b* = −0.01, *SE* = 0.02, 95% CI [−0.04, 0.02], and the intervention group, *b* = −0.00, *SE* = 0.01, 95% CI [−0.03, 0.02], was significant, see also Figure 4B.

### 2.4 Discussion

The aim of Study 1 was to assess the effect of the identity-reframing, background-specific strength intervention. As expected, there was a negative association between SES and social belonging in the control group, indicating that lower SES students feel less like a part of the student community. In line with our assumptions, the identity-reframing intervention could successfully buffer this effect, making the negative association disappear. This finding suggests that when low-SES students reflect on their past unique experiences and consider how this supports their academic journey, their background no longer undermines their sense of belonging.

As we expected, higher social belonging led to a higher academic self-concept. In total, we found a significant indirect path of SES via social belonging on academic self-concept for the control group. Importantly, this association was absent for the intervention group, showing that the intervention strengthens the self as a resource for low-SES students. We found the same pattern for fear of errors.

We explored whether the intervention could also function via academic identity. There was no association of SES with academic identity, either for the control group or students in the intervention group, suggesting that socioeconomic status does not influence the degree to which individuals perceive themselves as students. As a result, the intervention did not affect academic identity.

In summary, the first study demonstrated that an identity-reframing intervention focusing on background-specific strengths mitigated the negative association between SES and social belonging. Additionally, in the sense of social cure, we found that social belonging then positively impacts low-SES students’ academic self-concept and reduces their fear of errors in higher education. The study expands identity-reframing intervention on social belonging. Furthermore, we show that our brief version of the intervention is as effective as the version used in previous studies.

It is important to emphasize that none of our moderated mediation models showed a significant direct effect of SES on the outcome. Consequently, we characterize the indirect paths of the mediation model as a potential additional psychological mechanism rather than as an explanation for a direct effect.

## 3 Study 2

### 3.1 Introduction

In the second study, we examine a resource-strengthening, identity-reframing intervention for students with ADHD symptoms. Like the first study, the intervention draws on enhancing ingroup identification. By fostering students’ sense of belonging, we aim to improve academic engagement through the benefits of the social cure effect. We hereby try to establish social belonging as a vital resource for students with ADHD, who might lack other significant resources, such as a strong belief in their capabilities necessary to succeed in higher education (Sarid and Lipka, 2024). In line with Study 1, this study will investigate the impact of an identity-reframing intervention via social belonging on intrinsic motivation, an essential prerequisite for academic success (Trevino and DeFreitas, 2014). In Study 2, we did not directly measure social belonging. Instead, we focused on relatedness satisfaction, which strongly correlates with social belonging (Çıkrıkçı and Gençdoğan, 2022; Sheldon and Bettencourt, 2002). Therefore, in the sense of social cure, we expect that the intervention will lead to higher social belonging for students exhibiting ADHD symptoms (H2.1), which in turn is related to intrinsic motivation (H2.2) Consistent with the approach of the first study, we will exploratively examine whether the intervention fosters the academic identity of students with ADHD symptoms, which is then associated with increased intrinsic motivation.

### 3.2 Method

#### 3.2.1 Participants

We recruited 122 students with a mean age of *M* = 22.99 (*SD* = 4.16) via social media and e-mail lists. Regarding gender, 97 (79.51%) identified as female, 22 (18.03%) as male, two (1.64%) as diverse, and one (0.82%) did not state their gender. On average, students were in the fourth semester of their studies (*M* = 4.37, *SD* = 3.60). Twenty-one (17.21%) participants were diagnosed with ADHD.

#### 3.2.2 Measures

##### 3.2.2.1 ADHD symptoms

We assessed students’ ADHD Symptoms using a six-item scale based on the World Health Organization Adult ADHD Self-Report Scale (ASRS-5; Ustun et al., 2017). Participants answered the statements on a five-point Likert scale ranging from “never” to “very often” (e.g., “How often do you leave your seat in seminars or other courses where you actually have to stay seated?”). This scale serves as a screening tool for ADHD, where a score of 14 or above meets the DSM-V criteria. In the present research, we calculated a mean score to capture ADHD as a spectrum rather than using a categorical diagnosis (*α* = 0.73). The average score was *M* = 2.85 (*SD* = 0.73). We added four distractor items describing non-ADHD related behaviors at university to the scale, not to disclose the purpose of the study.

##### 3.2.2.2 Intrinsic motivation

We measured participants’ intrinsic motivation using the subscale “Interest” from the *Intrinsic Motivation Inventory* (IMI) (McAuley et al., 1989). The subscale consists of seven statements adapted to fit the academic context (e.g., “I enjoy studying”). Participants answered on a seven-point Likert scale ranging from “do not agree at all” to “completely agree.” It must be noted that only the subscale “interest” of the *Intrinsic Motivation Inventory* (IMI) measures intrinsic motivation per se; other scales are mostly predictors of intrinsic motivation (Center for Self-Determination Theory, n.d.). Thus, only the subscale “Interest” is used in subsequent analysis (α = 0.81).

##### 3.2.2.3 Relatedness satisfaction

We measured relatedness satisfaction using two items of the BPNSNF- Scale (Chen et al., 2015). Participants answered the statements on a seven-point Likert scale ranging from “do not agree at all” to “completely agree” (Items: “I feel that I am also important to the fellow students who mean something to me.,” “I have a warm feeling for the fellow students I spend time with.”). Both items correlated highly, *r* = 0.68, *p* < 0.001.

##### 3.2.2.4 Academic identity

We assessed academic identity with two items. Participants responded on a seven-point Likert scale ranging from “do not agree at all” to “completely agree” on how much they identified with their university (1) and being a student (2). Both items correlated highly, *r* = 0.51, *p* < 0.001.

#### 3.2.3 Procedure and design

After giving informed consent, participants responded to the statements regarding ADHD symptoms in the university context. We added distractor items to the scale so as not to disclose the aim of the study nor to influence responses to subsequent questions. Then, participants were randomly assigned to either the experimental or the control condition. In the experimental condition, participants were first instructed to list three strengths and explain how they could benefit their studies. Afterward, they were presented with a list of behaviors typically associated with students with ADHD that could hinder their academic progress or experience (e.g., “I sometimes find it difficult to plan efficiently and make good use of my time for my studies,” “I cannot sit still for several hours in a row and suppress my urge to move.”). Participants chose the three behaviors they could identify with the most. Following this, we showed students reframed versions of the statements they selected before (e.g., “I have a lot of resilience because, despite many setbacks, I continue to work on planning better and working more effectively.”, “Regular exercise boosts my energy and my imaginative thoughts, especially when I’m working on creative projects.”). Participants then indicated how much they agreed with each reframed statement. On the other hand, participants in the control condition were instructed to reflect on their everyday lives at university and note important aspects of it instead of thinking about their strengths. Afterward, they reviewed a list of common university behaviors and selected those that matched their experiences (e.g., “There are days when I take homemade snacks with me to university,” “I occasionally ask for feedback on my work to improve.”). We then assessed participants’ intrinsic motivation, need satisfaction, and frustration. Students stated their demographic information and their academic identity. We report only variables pertinent to this study’s research question.

#### 3.2.4 Analyses

To test whether the intervention indeed buffers the effect of ADHD symptoms on intrinsic motivation, we will use a moderated mediation model. The mean score of ADHD symptoms will be the independent variable, with the psychological resource of either academic identity or social belonging as the mediator. The path between ADHD symptoms and the resource will be moderated by the intervention. Intrinsic motivation is included as the dependent variable (for full effects, see Supplementary Tables 8, 11).

### 3.3 Results

For an overview of means, standard deviations, and correlations of the variables, see Table 3.

**Table 3:** Correlation matrix of the variables in Study 2.

Variable	*M*	*SD*	1	2	3
1. ADHD-Symptoms	2.85	0.73			
2. Relatedness Satisfaction	5.44	1.38	−0.08 [−0.25, 0.10]		
3. Academic Identity	4.90	1.25	0.09 [−0.09, 0.26]	0.37*** [0.21, 0.51]	
4. Intrinsic Motivation	5.11	0.97	−0.14 [−0.31, 0.04]	0.21* [0.03, 0.37]	0.41*** [0.25, 0.54]

*M* and *SD* represent mean and standard deviation, respectively. Values in square brackets indicate the 95% confidence interval for each correlation. *p < 0.05, ***p < 0.001.

#### 3.3.1 Relatedness satisfaction as the mediator

**Figure 5:**
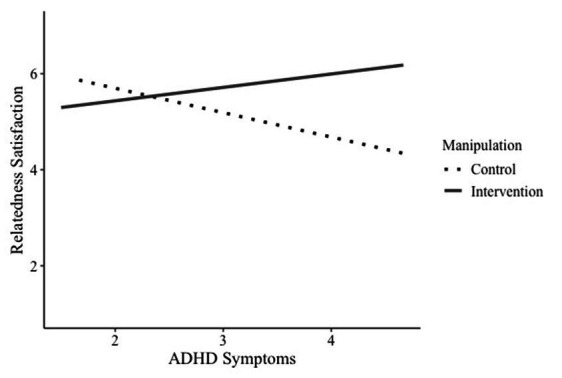
Interaction of manipulation and ADHD symptoms on relatedness satisfaction.

In the first moderated mediation model, we used ADHD symptoms as the independent variable, relatedness satisfaction as the mediator, and intrinsic motivation as the outcome variable. The path from ADHD symptoms to relatedness satisfaction was moderated by the intervention. There was a significant main effect of ADHD symptoms, *b* = −0.51, *SE* = 0.23, *p* = 0.030, 95% CI [−0.96, −0.52], but not for the intervention, *b* = 0.41, *SE* = 0.25, *p* = 0.010, 95% CI [−0.08, 0.98]. We found a significant interaction between ADHD symptoms and the intervention, *b* = 0.79, *SE* = 0.34, *p* = 0.022, 95% CI [0.11, 1.46], on relatedness satisfaction, indicating that the intervention modified the association between ADHD symptoms and relatedness satisfaction (H2.1). Analyses of the conditional effects of the moderation showed that for students in the control group, more ADHD symptoms were associated with less relatedness satisfaction, *b* = −0.51, *SE* = 0.23, *p* = 0.030, 95% CI [−0.96, −0.52]. For participants in the intervention group, this effect was not present, *b* = 0.28, *SE* = 0.25, *p* = 0.267, 95% CI [−0.22, 0.77], indicating that the intervention successfully buffered the effect of ADHD symptoms on relatedness satisfaction (see Figure 5). When we investigated the intervention using the Johnson-Neyman method, we found it to be especially beneficial for students reporting a high number of ADHD symptoms (see Supplementary Tables 9, 10).

**Figure 6:**
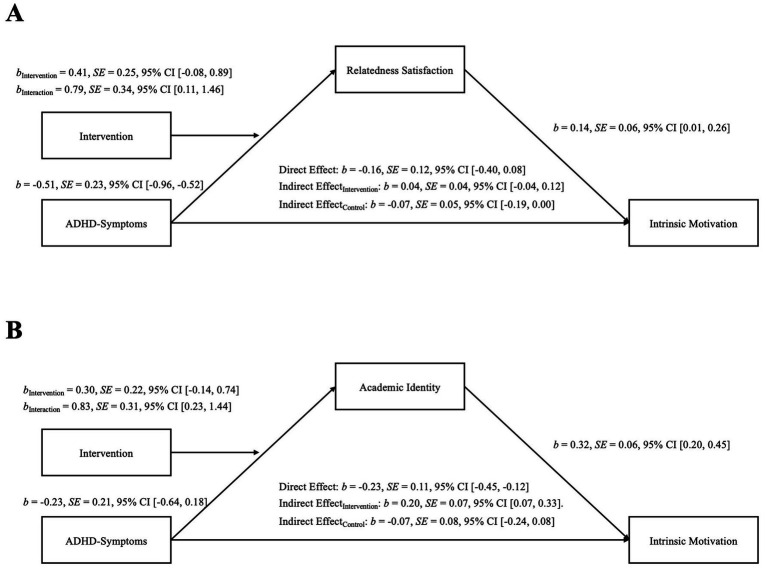
Moderated mediation models with **(A)** Relatedness satisfaction and **(B)** Academic identity as the mediators. *B*-values indicate unstandardized regression coefficients.

We found no significant direct effect of ADHD symptoms on intrinsic motivation, *b* = −0.16, *SE* = 0.12, *p* = 0.181, 95% CI [−0.40, 0.08]. However, intrinsic motivation was significantly associated with relatedness satisfaction, *b* = 0.14, *SE* = 0.06, *p* = 0.029, 95% CI [0.01, 0.26]. As such, we tested for indirect paths of ADHD symptoms on intrinsic motivation via relatedness satisfaction. We found that the more students’ need for relatedness was satisfied, the more intrinsic motivation they reported. However, there was no significant indirect path of ADHD symptoms via relatedness satisfaction on intrinsic motivation, either for the control group, *b* = −0.07, *SE* = 0.05, 95% CI [−0.19, 0.00], or the intervention group, *b* = 0.04, *SE* = 0.04, 95% CI [−0.04, 0.12]. The index of the moderated mediation further showed that there was no significant difference between the indirect effects of the control and intervention group, *b* = 0.11, *SE* = 0.07, 95% CI [−0.01, 0.26], see also Figure 6A. These findings suggest that although the intervention successfully buffers the effect of ADHD symptoms on relatedness satisfaction, there were no indirect paths via relatedness satisfaction on intrinsic motivation (H2.2).

#### 3.3.2 Academic identity as the mediator

**Figure 7:**
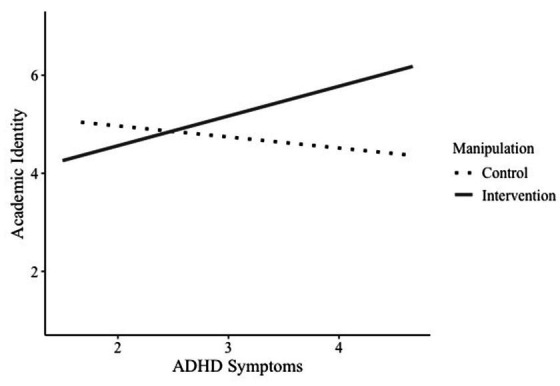
Interaction of manipulation and ADHD symptoms on academic identity.

In the second moderated mediation model, we kept the same structure from the previous model but exchanged relatedness satisfaction with academic identity. There were no significant main effects of ADHD symptoms, *b* = −0.23, *SE* = 0.21, *p* = 0.279, 95% CI [−0.64, 0.18], and the intervention on relatedness satisfaction, *b* = 0.30, *SE* = 0.22, *p* = 0.176, 95% CI [−0.14, 0.74]. We found a significant interaction between ADHD symptoms and intervention on academic identity, *b* = 0.83, *SE* = 0.31, *p* = 0.008, 95% CI [0.23, 1.44]. Conditional analyses of the intervention showed that in the control group, ADHD symptoms and academic identity were not significantly related, *b* = −0.22, *SE* = 0.21, *p* = 0.279, 95% CI [−0.64, 0.18]. However, when students in the experimental condition saw reframed statements about ADHD behaviors, we found a positive association between ADHD symptoms and academic identity, *b* = 0.61, *SE* = 0.23, *p* = 0.008, 95% CI [0.17, 1.05] (Figure 7). When analyzing the interaction using the Johnson-Neyman method, we discovered that the intervention was most effective for students exhibiting a high number of ADHD symptoms (see Supplementary Tables 12, 13).

In addition, there was a significant direct effect of ADHD symptoms on intrinsic motivation, *b* = −0.23, *SE* = 0.11, *p* = 0.039, 95% CI [−0.45, −0.12]. The more symptoms they reported, the less intrinsic motivation students indicated. Furthermore, academic identity was significantly associated with intrinsic motivation, *b* = 0.32, *SE* = 0.06, *p* < 0.001, 95% CI [0.20, 0.45], indicating that students who identified as students also reported more intrinsic motivation for their studies. Looking at the indirect effects of ADHD symptoms via academic identity on intrinsic motivation, we found no effect for the control group, *b* = −0.07, *SE* = 0.08, 95% CI [−0.24, 0.08]. However, we found a significant indirect effect via academic identity for the experimental group, *b* = 0.20, *SE* = 0.07, 95% CI [0.07, 0.33], see also Figure 6B. This suggests that the intervention increases intrinsic motivation through academic identity. The index of the moderated mediation shows that the indirect effects for the control and intervention groups differ significantly, *b* = 0.27, *SE* = 0.11, 95% CI [0.08, 0.49].

### 3.4 Discussion

In summary, we find that the intervention, which focuses on strengths and reframing ADHD behavior, significantly impacts the relationship between ADHD symptoms and relatedness satisfaction. The intervention neutralizes the negative impact of ADHD symptoms on relatedness satisfaction. Thus, by reframing stigmatized ADHD behaviors, students reported higher relatedness satisfaction. However, there was no indirect path of ADHD symptoms on intrinsic motivation via relatedness satisfaction.

Additionally, focusing on strengths and reframing ADHD behavior even led to a positive association between symptoms and academic identity. We found a significant indirect effect via academic identity on intrinsic behavior, indicating that empowering the academic self through intervention increases intrinsic motivation, using academic identity as a psychological resource. This study takes a novel approach by applying identity-reframing interventions to students with ADHD symptoms. It demonstrates that deriving strengths from prior deficit narratives can benefit various disadvantaged groups in higher education.

## 4 General discussion

Students need to draw from various resources to pursue a degree in higher education. One vital resource is belonging to a relevant group in the university setting. According to the social cure approach, group membership leads to more well-being, which in turn can enhance academic engagement. However, students from disadvantaged groups, such as those with low socioeconomic backgrounds or ADHD symptoms, often feel misplaced in higher education and thus lack social belonging as a crucial resource. In the present research, we aimed to test resource-strengthening, identity-reframing interventions focusing on reframing deficit narratives for those two vulnerable student groups.

In the first study, we demonstrated that an intervention focusing on the background-specific strengths of low-SES students mitigated the detrimental association of low SES on students’ social belonging. This means that by reflecting on the challenges they have mastered and the strengths that arise from these experiences, low SES students exhibit a greater sense of social belonging with their peers in higher education. Additionally, we found that the intervention alleviated the negative path from SES to academic self-concept and fear of errors through social belonging. Notably, this effect was not present when analyzing academic identity as a mediator instead of social belonging, stating that feeling as part of a group is a vital resource for students from low socioeconomic backgrounds.

In the second study, we focused on a reframing intervention for students with ADHD symptoms. We found that reframing ADHD-related behavior, which is often depicted as hindering academic success, buffers the negative relationship between ADHD symptoms and relatedness satisfaction – a proxy for social belonging. However, that sense of belonging was not related to intrinsic motivation concerning participants’ studies. Interestingly, academic identity successfully alleviated the relationship between ADHD symptoms and intrinsic motivation, indicating that strengthening participants’ perception as capable students, even with ADHD, seems to be supportive regarding their academic engagement.

### 4.1 Theoretical contribution

#### 4.1.1 Social cure

In the current research, we successfully demonstrated the social cure effect for disadvantaged groups in higher education. Group membership emerges as a vital resource for both low-SES students and those with ADHD symptoms. Interestingly, there is a difference concerning the role of academic identity between those two student groups. For low-SES students, the buffering effect was only present for social belonging, not for academic identity. This may indicate that being a student from a low socioeconomic background may have more implications on whether a student feels integrated within the community. While they may struggle with belonging to their peers, their background seems not to impact their perception of themselves as students. Thus, low-SES students might not fit in with their peers but do view themselves as capable students.

This might be different for students with ADHD symptoms. Here, academic identity plays a much more pivotal role, buffering the detrimental effect of ADHD symptoms on intrinsic motivation. This might be due to the different nature of disadvantage compared to low-SES students. ADHD directly affects learning and performance in academic settings, resulting in them questioning their abilities more than their belonging to the student community. For low-SES, this might be the other way around. Lack of familiarity with university culture might lead to doubting one’s place in the community but not necessarily one’s abilities and, thus, academic identity. However, additional research is necessary to understand further the different mechanisms of social cure for both groups.

#### 4.1.2 Rejection identification model

Social cure offers a valuable framework to explain why and how social identification with a group benefits well-being, particularly for individuals from disadvantaged backgrounds. However, belonging to underprivileged groups also comes with psychological costs, such as prejudice and discrimination (Schmitt and Branscombe, 2002). This is also true for both groups investigated in this manuscript: students from low SES backgrounds and those with ADHD symptoms. The Rejection Identification Model (RIM) provides an approach to understand why even members of disadvantaged and stigmatized groups benefit from ingroup identification, as discrimination fosters ingroup identification, which in turn acts as a protective factor to mitigate the detrimental effects of discrimination. There is a substantial body of literature showing this effect for different disadvantaged groups: people with disabilities, multiracial people, ethnic minorities (Bogart et al., 2018; Giamo et al., 2012; Tabbah et al., 2016). Our findings support the initial assumptions of RIM: Firstly, students from low SES backgrounds and those with ADHD symptoms should distance themselves to escape the stigma associated with their group in higher education. However, in our studies, their sense of social belonging improves by focusing on the strengths developed through facing unique challenges. For low-SES students, RIM would suggest that although these students may feel out of place within the academic community, that experience can reinforce their identification with others from similar socioeconomic backgrounds. These students may then derive strength and a sense of belonging from the experiences they have in common with their ingroup members. Instead of trying to fit in with their neurotypical peers, which may only increase feelings of inadequacy and reinforce their sense of social belonging threat, these students may benefit from stronger identification with others who share their neurodivergent identity. However, in the present research, we show that by reframing the deficit narratives, students from low SES and those with ADHD symptoms even reported more general social belonging, acting as a psychological resource in the sense of social cure. This extends RIM, which would assume that only identification with the disadvantaged ingroup would serve as a protective factor.

#### 4.1.3 Identity-reframing intervention

The present studies further extend research on identity-reframing interventions. These types of social psychological interventions focus on multiple outcomes, such as the academic achievement of low SES and refugee students, as well as the confidence and goal pursuit of individuals with depression (Bauer et al., 2021, 2024, 2025). However, investigating the effects of identity-reframing interventions on social belonging and academic identity is novel. This provides additional evidence that deficit narratives are detrimental not only to academic and health outcomes but also hinder the satisfaction of basic human needs, such as the need to belong. Another contribution to the literature on background-specific strengths is the group of ADHD students. A growing body of literature focuses on the individual struggles of these students, such as their lack of organizational skills and difficulties navigating academic life in general. The present paper extends this research by reframing these often negatively portrayed aspects, thereby changing the narrative of being an ADHD student in higher education. Moreover, we demonstrate that identity-reframing interventions are effective even when skipping an introduction and the presentation of success stories from disadvantaged group members. Therefore, all students, whether they belong to a disadvantaged group or not, can participate in the intervention.

#### 4.1.4 Contribution to COR

Studying is a process and a life phase that requires various resources (Stelnicki et al., 2015). The Conservation of Resource Theory postulates four types of resources that are essential for individuals (Hobfoll, 1989): objects, conditions, personal characteristics, and energies. In the present paper, we investigated two student groups that lack resources, thus making studying much more challenging. Low SES may miss key object resources, such as technical equipment or appropriate housing (Broton and Goldrick-Rab, 2018; Reisdorf et al., 2020); ADHD students experience limitations in their personal resources, such as their restricted attention and subsequent struggles in exams, for example (Advokat et al., 2011). Both groups unite that, due to their lack of resources, a shared resource is also depleted: their sense of belonging at university (Godfrey-Harris and Shaw, 2023; Jetten et al., 2008). We contribute to COR literature by examining how restoring a lacking resource, in this case, social belonging, can help buffer the absence of other resources. In our view, this is very much in line with the second principle of COR: “Resource investment principle. People must invest resources in order to protect against resource loss, recover from losses, and gain resources” (Hobfoll et al., 2018, p. 106). If we support vulnerable groups in higher education through identity-reframing interventions and an increased sense of social belonging, they are better protected against resource loss stemming from their backgrounds, such as socioeconomic status and ADHD symptoms.

### 4.2 Practical implications

Our findings also allow us to draw implications for higher education practice. Students from vulnerable groups or those with deficit narratives often comprise the target group for support programs in higher education, such as coaching, mentoring, or other onboarding activities (Valentine et al., 2011). However, to effectively support members of these student groups, our research suggests that it might be more beneficial to foster their sense of belonging rather than solely providing concrete strategies for navigating everyday life at university. This seems especially evident for students with ADHD. Anastopoulos and King (2015) show that cognitive behavior therapy in combination with a mentoring program led to an increase in academic success. However, the intervention focused on psychoeducational content behavioral strategies and did not address the narrative around ADHD. Based on our findings, adding identity-reframing modules could strengthen such an initiative. Thomas (2014) explicitly points out that although universities offer programs for low SES students, they typically stem from a deficit perception of those students. The identity-reframing intervention offers a novel approach by altering the narrative surrounding the commonly labeled weaknesses. Specifically, encouraging interaction around what low SES students have already accomplished and how these experiences can assist them in higher education can be a crucial aspect of any support initiative.

### 4.3 Limitations and future research

Some limitations to our research should also be noted. A crucial point that makes comparing studies one and two difficult is the different operationalization of the constructs. In study one, we used the social belonging and academic identity scale by Möller et al. (2022). However, in the second study, we used items initially based on the need for relatedness to access social belonging and operationalized academic identity with different items than in study one. To make robust comparisons between the interventions for low-SES and students with ADHD symptoms, all constructs should be accessed using the same instruments. However, we can acknowledge that, despite the differences in measurement, comparisons can be made between both studies. Comparing the effect of identity-reframing interventions on low SES students, a well-researched group in this context, with students exhibiting ADHD symptoms, is novel and a strength of the present paper.

Further, we measured different dependent variables. In study one, we focused on academic self-concept and fear of errors; in the second study, the outcome was intrinsic motivation. Although all three variables are closely connected to academic engagement, a uniform measure would be helpful. Nonetheless, the multi-faceted approach in the present studies demonstrates that identity-reframing interventions for low SES students affect not only academic performance and grades (Bauer et al., 2025) but also deepen the understanding of the underlying psychological mechanisms that range from motivational to cognitive concepts.

In addition, it is important to highlight that the first study did not find a direct association between SES and both academic self-concept and fear of errors. This rightfully questions the generalizability of the effect. However, we did observe an indirect effect of SES on academic self-concept and fear of errors through social belonging. Notably, this indirect association was reduced by the identity reframing intervention and its impact on social belonging.

Moreover, we did not account for the different institutions of higher education. Besides traditional universities, students can also attend vocational universities or universities of applied sciences that might differ in student demographics and educational structure. Traditional universities typically attract students from higher SES and academic backgrounds, whereas universities of applied sciences often have a more diverse educational and socioeconomic student body (Institut für Höhere Studien, 2024). Therefore, low-SES students might not be struggling as much with social belonging at those tertiary institutions. Furthermore, universities of applied sciences often have a structure that resembles a high school, with fixed classes and schedules. The structured environment might be especially beneficial for students with ADHD symptoms, who would feel more integrated within the student community, as the consistent structure better fits their additional needs regarding organization and self-management. Nevertheless, it has to be noted that in Austria almost 66% (Institut für Höhere Studien, 2024) of students, so a vast majority, attend traditional universities, allowing us to generalize our findings to the broad student population.

Additionally, expanding the investigated groups would be necessary to fully understand the social cure effect of these identity-reframing interventions. Next to students from low-SES backgrounds and students with ADHD symptoms, many more groups of students face additional challenges and hurdles in higher education. One possible group could be first-generation students. This group is often depicted as disadvantaged in the academic setting due to a lack of knowledge of university culture and norms (Soria and Stebleton, 2012) with lower social belonging compared to their peers from academic households (Pedler et al., 2022). Here, an identity-reframing, background-specific strength intervention could be beneficial as First-Generation students can be seen as assertive, resilient, and maybe even more open to innovative strategies that deviate from the status quo.

## 5 Conclusion

Taken together, this research aimed to foster academic engagement among members of disadvantaged groups, namely students with low-SES backgrounds and ADHD symptoms, by using brief social-psychological interventions that focus on reframing deficit narratives and thus increasing social belonging. In the present paper, we successfully combine different approaches to understand and support vulnerable student populations. We demonstrate that studying, in the sense of COR framework, is a resource-based process and that a lack of resources can be effectively counteracted. Furthermore, we highlight how identity-reframing interventions can enhance social belonging and enable it to become a resource for these students. Aligning with the social cure perspective, we provide evidence that social belonging is essential for students from disadvantaged backgrounds to overcome obstacles in higher education. Practical implications can be drawn for designing interventions like coaching and mentoring that foster social belonging by helping students reframe their past challenges into strengths. Our research aims to encourage further development of additional interventions to benefit numerous students who face additional challenges with their university degrees. Future research may explore extending identity-reframing interventions to other student groups dealing with unique challenges in higher education, such as adult learners, students with children, or those with disabilities.

## Data Availability

Data and code for Studies 1 and 2 are available under the following link: https://osf.io/654kt/?view_only=734c43225adc4ac8ba6b27a61e4b3aa0.

## Footnotes

^1^Data and code for Studies 1 and 2 are available under the following link: https://osf.io/654kt/?view_only=734c43225adc4ac8ba6b27a61e4b3aa0
