# Supplementary material for: Strengthening resources through identity-reframing interventions: empowerment for students with low socioeconomic status and with ADHD symptoms
Source: Front Psychol. 2025 Jun 25;16:1525850. doi: 10.3389/fpsyg.2025.1525850 (PMC12238953; doi:10.3389/fpsyg.2025.1525850)
Supplement: Supplementary file 1 [file Data_Sheet_1.docx]

Supplementary Material

| Item | Factor Loading | |
| --- | --- | --- |
|  | 1 | 2 |
| Factor 1: Academic Identity |  |  |
| 1. I can identify well with my studies | **0.65** | 0.14 |
| 1. I like being a student at this university. | **0.68** |  |
| 1. I have trouble navigating the academic environment. (-) | -0.33 | -0.39 |
| 1. I would recommend my studies to others. | **0.67** |  |
| 1. I often feel like I don’t really belong at the university. (-) | -0.41 | -0.47 |
| 1. My expectations of my studies at this university were fully met. | **0.70** |  |
| 1. Sometimes I wonder if studying is right for me. (-) | **-0.49** | -0.17 |
| Factor 2: Social Belonging |  |  |
| 1. I (already) have a lot of good contacts with the instructors at my department. | 0.17 | **0.47** |
| 1. I already have many good contacts with the other students at my department |  | **0.86** |
| 1. I have already become very accustomed to studying. | 0.49 | 0.50 |
| 1. I have (already) made friends at university. |  | **0.83** |
| 1. I feel (already) in good hands in my studies | 0.57 | 0.63 |

# Factor Analysis Study 1

**Table 1**

*Factor Analysis for the Scales Academic Identity and Social Belonging*

*Note. N =* 270. The extraction method was Maximum Likelihood Factoring. Values in bold depict the factor to which the item was assigned. Items with no bold values were dropped. Reverse-scored items are denoted with (-).

# Full Effects for Moderated Mediation Models Study 1

**Table 2**

*Moderated Mediation Model for Social Belonging as the Mediator and Academic Self-Concept as the Dependent Variable*

|  | Mediator Variable Social Belonging | | | | |
| --- | --- | --- | --- | --- | --- |
|  | *b* | *SE* | *t* | *p* | 95 % CI |
| SES | 0.24 | 0.07 | 3.41 | .001 | [0.10, 0.38] |
| Intervention | 0.00 | 0.15 | 0.03 | .980 | [-0.30, 0.30] |
| SES x Intervention | -0.18 | 0.09 | -1.96 | .051 | [-0.36, 0.00] |
| Conditional effects of the Moderator |  |  |  |  |  |
| Control Group | 0.24 | 0.07 | 3.41 | .001 | [0.10, 0.38] |
| Intervention Group | 0.06 | 0.06 | 1.00 | .361 | [-0.06, 0.18] |
|  | Dependent Variable Academic Self-Concept | | | | |
| SES | -0.00 | 0.03 | -0.12 | .907 | [-0.06, 0.05] |
| Social Belonging | 0.14 | 0.04 | 3.79 | <.001 | [0.07, 0.21] |
| Conditional indirect effects |  |  |  |  |  |
| Control Group | 0.03 | 0.01 |  |  | [0.01, 0.06] |
| Intervention Group | 0.01 | 0.01 |  |  | [-0.01, 0.03] |

*Note.* B-values represent unstandardized regression coefficients. CI = Confidence Interval

|  | Mediator Variable Social Belonging | | | | |
| --- | --- | --- | --- | --- | --- |
|  | *b* | *SE* | *t* | *p* | 95 % CI |
| SES | 0.24 | 0.07 | 3.41 | .001 | [0.10, 0.38] |
| Intervention | 0.00 | 0.15 | 0.03 | .980 | [-0.30, 0.30] |
| SES x Intervention | -0.18 | 0.09 | -1.96 | .051 | [-0.36, 0.00] |
| Conditional effects of the Moderator |  |  |  |  |  |
| Control Group | 0.24 | 0.07 | 3.41 | .001 | [0.10, 0.38] |
| Intervention Group | 0.06 | 0.06 | 1.00 | .361 | [-0.06, 0.18] |
|  | Dependent Variable Fear of Errors | | | | |
| SES | -0.01 | 0.05 | -0.26 | .795 | [-0.11, 0.08] |
| Social Belonging | -0.24 | 0.06 | -3.80 | .001 | [-0.37, -0.12] |
| Conditional indirect effects |  |  |  |  |  |
| Control Group | -0.06 | 0.02 |  |  | [-0.11, -0.02] |
| Intervention Group | -0.01 | 0.02 |  |  | [-0.05, 0.02] |

**Table 3**

*Moderated Mediation Model for Social Belonging as the Mediator and Fear of Errors as the Dependent Variable*

*Note.* B-values represent unstandardized regression coefficients. CI = Confidence Interval

**Table 4**

*Conditional Effects for the Interaction of SES and Intervention on Social Belonging by Levels of SES*

| SES | *b* | *SE* | *t* | *p* | 95 % CI |
| --- | --- | --- | --- | --- | --- |
| -1.66 | 0.31 | 0.22 | 1.41 | .160 | [-0.12, 0.73] |
| 0.00 | 0.00 | 0.15 | 0.03 | .980 | [-0.30, 0.30] |
| 1.66 | -0.30 | 0.22 | -1.38 | .170 | [-0.73, 0.13] |

*Note.* B-values represent unstandardized regression coefficients. CI = Confidence Interval

**Table 5**

*Johnson-Neyman Intervals for the Interaction of SES and Intervention on Social Belonging by Levels of SES*

| SES | *b* | *SE* | *t* | *p* | 95% CI |
| --- | --- | --- | --- | --- | --- |
| -5.23 | 0.95 | 0.51 | 1.87 | .062 | [-0.05, 1.96] |
| -4.80 | 0.88 | 0.47 | 1.86 | .064 | [-0.05, 1.80] |
| -4.37 | 0.80 | 0.43 | 1.84 | .067 | [-0.06, 1.65] |
| -3.94 | 0.72 | 0.40 | 1.81 | .071 | [-0.06, 1.50] |
| -3.51 | 0.64 | 0.36 | 1.78 | .076 | [-0.07, 1.35] |
| -3.08 | 0.56 | 0.32 | 1.74 | .083 | [-0.08, 1.20] |
| -2.65 | 0.49 | 0.29 | 1.68 | .095 | [-0.09, 1.06] |
| -2.23 | 0.41 | 0.26 | 1.59 | .113 | [-0.10, 0.91] |
| -1.80 | 0.33 | 0.23 | 1.46 | .145 | [-0.12, 0.78] |
| -1.37 | 0.25 | 0.20 | 1.27 | .205 | [-0.14, 0.64] |
| -0.94 | 0.17 | 0.18 | 0.99 | .321 | [-0.17, 0.52] |
| -0.51 | 0.10 | 0.16 | 0.61 | .545 | [-0.22, 0.41] |
| -0.08 | 0.02 | 0.15 | 0.12 | .901 | [-0.28, 0.32] |
| 0.35 | -0.06 | 0.16 | -0.38 | .706 | [-0.37, 0.25] |
| 0.77 | -0.14 | 0.17 | -0.81 | .418 | [-0.47, 0.19] |
| 1.20 | -0.21 | 0.19 | -1.14 | .257 | [-0.59, 0.16] |
| 1.63 | -0.29 | 0.21 | -1.36 | .174 | [-0.72, 0.13] |
| 2.06 | -0.37 | 0.24 | -1.52 | .131 | [-0.85, 0.11] |
| 2.49 | -0.45 | 0.28 | -1.62 | .106 | [-0.99, 0.10] |
| 2.92 | -0.53 | 0.31 | -1.69 | .092 | [-1.14, 0.09] |
| 3.35 | -0.60 | 0.35 | -1.75 | .082 | [-1.29, 0.08] |
| 3.77 | -0.68 | 0.38 | -1.78 | .076 | [-1.43, 0.07] |

*Note.* B-values represent unstandardized regression coefficients. CI = Confidence Interval

**Table 6**

*Moderated Mediation Model for Academic Identity as the Mediator and Academic Self-Concept as the Dependent Variable*

|  | Mediator Variable Academic Identity | | | | |
| --- | --- | --- | --- | --- | --- |
|  | *b* | *SE* | *t* | *p* | 95 % CI |
| SES | 0.02 | 0.05 | 0.41 | .685 | [-0.07, 0.12] |
| Intervention | 0.04 | 0.11 | 0.41 | .682 | [-0.17, 0.26] |
| SES x Intervention | -0.01 | 0.07 | -0.12 | .908 | [-0.14, 0.12] |
|  | Dependent Variable Academic Self-Concept | | | | |
| SES | 0.01 | 0.03 | 0.40 | .692 | [-0.04, 0.06] |
| Academic Identity | 0.32 | 0.05 | 6.50 | <.001 | [0.22, 0.42] |
| Conditional indirect effects |  |  |  |  |  |
| Control Group | 0.01 | 0.02 |  |  | [-0.02, 0.04] |
| Intervention Group | 0.00 | 0.01 |  |  | [-0.02, 0.03] |

*Note.* B-values represent unstandardized regression coefficients. CI = Confidence Interval

**Table 7**

*Moderated Mediation Model for Academic Identity as the Mediator and Fear of Errors as the Dependent Variable*

|  | Mediator Variable Academic Identity | | | | |
| --- | --- | --- | --- | --- | --- |
|  | *b* | *SE* | *t* | *p* | 95 % CI |
| SES | 0.02 | 0.05 | 0.41 | .685 | [-0.07, 0.12] |
| Intervention | 0.04 | 0.11 | 0.41 | .682 | [-0.17, 0.26] |
| SES x Intervention | -0.01 | 0.07 | -0.12 | .908 | [-0.14, 0.12] |
|  | Dependent Variable Fear of Errors | | | | |
| SES | -0.04 | 0.05 | -0.84 | .399 | [-0.14, 0.05] |
| Academic Identity | -0.30 | 0.09 | -3.27 | .001 | [-0.48, -0.12] |
| Conditional indirect effects |  |  |  |  |  |
| Control Group | -0.01 | 0.01 |  |  | [-0.04, 0.02] |
| Intervention Group | 0.00 | 0.01 |  |  | [-0.03, 0.02] |

*Note.* B-values represent unstandardized regression coefficients. CI = Confidence Interval

# Full Effects for Moderated Mediation Models Study 2

**Table 8**

*Moderated Mediation Model for Relatedness Satisfaction as the Mediator and Intrinsic Motivation as the Dependent Variable*

|  | Mediator Variable Relatedness Satisfaction | | | | |
| --- | --- | --- | --- | --- | --- |
|  | *b* | *SE* | *t* | *p* | 95 % CI |
| ADHD-Symptoms | -0.51 | 0.23 | -2.20 | .030 | [-0.96, -0.05] |
| Intervention | 0.41 | 0.25 | 1.65 | .102 | [-0.08, 0.89] |
| ADHD-Symptoms x Intervention | 0.79 | 0.34 | 2.31 | .022 | [0.11, 1.46] |
|  | Dependent Variable Intrinsic Motivation | | | | |
| ADHD-Symptoms | -0.16 | 0.12 | -1.35 | .181 | [-0.40, 0.08] |
| Relatedness Satisfaction | 0.14 | 0.06 | 2.21 | .029 | [0.01, 0.26] |
| Conditional indirect effects |  |  |  |  |  |
| Control Group | -0.07 | 0.05 |  |  | [-0.18, 0.00] |
| Intervention Group | 0.04 | 0.04 |  |  | [-0.04, 0.12] |

*Note.* B-values represent unstandardized regression coefficients. CI = Confidence Interval

**Table 9**

| *ADHD Symptoms* | *b* | *SE* | *t* | *p* | 95 % CI |
| --- | --- | --- | --- | --- | --- |
| -0.73 | -0.16 | 0.35 | -0.47 | .638 | [-0.85, 0.52] |
| 0.00 | 0.41 | 0.25 | 1.65 | .102 | [-0.08, 0.89] |
| 0.73 | 0.98 | 0.35 | 2.80 | .006 | [0.29, 1.67] |

*Conditional Effects for the Interaction of ADHD Symptoms and Intervention on Relatedness Satisfaction by Levels of ADHD Symptoms*

*Note.* B-values represent unstandardized regression coefficients. CI = Confidence Interval

**Table 10**

*Johnson-Neyman Intervals for the Interaction of ADHD Symptoms and Intervention on Relatedness Satisfaction by Levels of ADHD Symptoms*

| ADHD Symptoms | *b* | *SE* | *t* | *p* | 95% CI |
| --- | --- | --- | --- | --- | --- |
| -1.35 | -0.65 | 0.52 | -1.26 | .210 | [-1.68, 0.37] |
| -1.19 | -0.53 | 0.47 | -1.12 | .265 | [-1.47, 0.41] |
| -1.03 | -0.41 | 0.43 | -0.95 | .346 | [-1.25, 0.44] |
| -0.87 | -0.28 | 0.39 | -0.73 | .468 | [-1.04, 0.48] |
| -0.72 | -0.16 | 0.35 | -0.45 | .652 | [-0.84, 0.53] |
| -0.56 | -0.03 | 0.31 | -0.10 | .919 | [-0.65, 0.58] |
| -0.40 | 0.09 | 0.28 | 0.33 | .741 | [-0.46, 0.65] |
| -0.24 | 0.22 | 0.26 | 0.84 | .403 | [-0.30, 0.73] |
| -0.08 | 0.34 | 0.25 | 1.38 | .170 | [-0.15, 0.83] |
| 0.08 | 0.47 | 0.25 | 1.88 | .062 | [-0.02, 0.96] |
| 0.11 | 0.49 | 0.25 | 1.98 | .050 | [0.00, 0.99] |
| 0.24 | 0.59 | 0.26 | 2.28 | .024 | [0.08, 1.11] |
| 0.39 | 0.72 | 0.28 | 2.55 | .012 | [0.16, 1.27] |
| 0.55 | 0.84 | 0.31 | 2.71 | .008 | [0.23, 1.46] |
| 0.71 | 0.97 | 0.35 | 2.79 | .006 | [0.28, 1.65] |
| 0.87 | 1.09 | 0.39 | 2.83 | .006 | [0.33, 1.85] |
| 1.03 | 1.21 | 0.43 | 2.83 | .005 | [0.37, 2.06] |
| 1.19 | 1.34 | 0.47 | 2.83 | .006 | [0.40, 2.28] |
| 1.34 | 1.46 | 0.52 | 2.81 | .006 | [0.43, 2.49] |
| 1.50 | 1.59 | 0.57 | 2.79 | .006 | [0.46, 2.71] |
| 1.66 | 1.71 | 0.62 | 2.77 | .006 | [0.49, 2.94] |
| 1.82 | 1.84 | 0.67 | 2.75 | .007 | [0.52, 3.16] |

*Note.* B-values represent unstandardized regression coefficients. CI = Confidence Interval

**Table 11**

|  | Mediator Variable Academic Identity | | | | |
| --- | --- | --- | --- | --- | --- |
|  | *b* | *SE* | *t* | *P* | 95 % CI |
| ADHD-Symptoms | -0.23 | 0.21 | -1.09 | .279 | [-0.64, 0.18] |
| Intervention | 0.30 | 0.22 | 1.36 | .176 | [-0.14, 0.74] |
| ADHD-Symptoms x Intervention | 0.83 | 0.31 | 2.72 | .008 | [0.23, 1.44] |
| Conditional effects of the Moderator |  |  |  |  |  |
| Control Group | -0.23 | 0.21 | -1.09 | .279 | [-0.64, 0.18] |
| Intervention Group | 0.61 | 0.23 | 2.70 | .008 | [0.16, 1.05] |
|  | Dependent Variable Intrinsic Motivation | | | | |
| ADHD-Symptoms | -0.23 | 0.11 | -2.09 | .039 | [-0.45, -0.01] |
| Academic Identity | 0.32 | 0.06 | 5.09 | <.001 | [0.20, 0.45] |
| Conditional indirect effects |  |  |  |  |  |
| Control Group | -0.07 | 0.08 |  |  | [-0.24, 0.08] |
| Intervention Group | 0.20 | 0.07 |  |  | [0.07, 0.34] |

*Moderated Mediation Model for Academic Identity as the Mediator and Intrinsic Motivation as the Dependent Variable*

*Note.* B-values represent unstandardized regression coefficients. CI = Confidence Interval

**Table 12**

*Conditional Effects for the Interaction of ADHD Symptoms and Intervention on Academic Identity by Levels of ADHD Symptoms*

| *ADHD Symptoms* | *b* | *SE* | *t* | *p* | 95 % CI |
| --- | --- | --- | --- | --- | --- |
| -0.73 | -0.30 | 0.31 | -0.96 | .337 | [-0.92, 0.32] |
| 0.00 | 0.30 | 0.22 | 1.36 | .176 | [-0.14, 0.74] |
| 0.73 | 0.91 | 0.31 | 2.88 | .005 | [0.28, 1.53] |

*Note.* B-values represent unstandardized regression coefficients. CI = Confidence Interval

**Table 13**

*Johnson-Neyman Intervals for the Interaction of ADHD Symptoms and Intervention on Academic Identity by Levels of ADHD Symptoms*

| *ADHD Symptoms* | *b* | *SE* | *t* | *p* | 95 % CI |
| --- | --- | --- | --- | --- | --- |
| -1.35 | -0.82 | 0.47 | -1.76 | .082 | [-1.75, 0.11] |
| -1.19 | -0.69 | 0.43 | -1.62 | .108 | [-1.53, 0.15] |
| -1.03 | -0.56 | 0.38 | -1.45 | .151 | [-1.32, 0.21] |
| -0.87 | -0.42 | 0.35 | -1.23 | .222 | [-1.11, 0.26] |
| -0.72 | -0.29 | 0.31 | -0.94 | .347 | [-0.91, 0.32] |
| -0.56 | -0.16 | 0.28 | -0.58 | .564 | [-0.71, 0.39] |
| -0.40 | -0.03 | 0.25 | -0.12 | .907 | [-0.53, 0.47] |
| -0.24 | 0.10 | 0.23 | 0.44 | .662 | [-0.36, 0.56] |
| -0.08 | 0.23 | 0.22 | 1.05 | .296 | [-0.21, 0.68] |
| 0.08 | 0.37 | 0.22 | 1.64 | .104 | [-0.08, 0.81] |
| 0.18 | 0.45 | 0.23 | 1.98 | .050 | [0.00, 0.91] |
| 0.24 | 0.50 | 0.23 | 2.13 | .035 | [0.04, 0.96] |
| 0.39 | 0.63 | 0.25 | 2.49 | .014 | [0.13, 1.13] |
| 0.55 | 0.76 | 0.28 | 2.72 | .007 | [0.21, 1.31] |
| 0.71 | 0.89 | 0.31 | 2.87 | .005 | [0.28, 1.51] |
| 0.87 | 1.02 | 0.35 | 2.95 | .004 | [0.34, 1.71] |
| 1.03 | 1.16 | 0.39 | 3.00 | .003 | [0.39, 1.92] |
| 1.19 | 1.29 | 0.43 | 3.02 | .003 | [0.44, 2.13] |
| 1.34 | 1.42 | 0.47 | 3.03 | .003 | [0.49, 2.35] |
| 1.50 | 1.55 | 0.51 | 3.04 | .003 | [0.54, 2.56] |
| 1.66 | 1.68 | 0.56 | 3.03 | .003 | [0.58, 2.78] |
| 1.82 | 1.81 | 0.60 | 3.03 | .003 | [0.63, 3.00] |

*Note.* B-values represent unstandardized regression coefficients. CI = Confidence Interval
